# Prognostic value of log odds of positive lymph nodes, lymph node ratio, and *N* stage in patients with colorectal signet ring cell carcinoma: A retrospective cohort study

**DOI:** 10.3389/fsurg.2022.1019454

**Published:** 2023-01-05

**Authors:** Xing Hu, Lixin Jiang, Jingzhou Wu, Weida Mao

**Affiliations:** Department of General Surgery, Jiangyin Hospital of Traditional Chinese Medicine, Wuxi, China

**Keywords:** log odds of positive lymph nodes, lymph node ratio, pN stage, colorectal signet ring cell carcinoma, SEER

## Abstract

**Aim:**

Little attention has been paid in the prognosis of colorectal signet ring cell carcinoma (SRCC). This study aims to explore the predictive capacity of log odds of positive lymph nodes (LODDS), lymph node ratio (LNR), and pN stage in the prognosis of patients with colorectal SRCC.

**Methods:**

A retrospective cohort study was designed, and data were extracted from the Surveillance, Epidemiology and End Results (SEER) database. Data on demographic characteristics, clinicopathological features, and treatment were extracted. Outcomes were overall survival (OS) and cancer-specific survival (CSS). Association of LODDS, LNR, and pN stage with OS and CSS were explored using Cox proportional hazard model and Cox competing risk model, respectively, with results showing as hazard ratio and 95% confidence interval (CI). Predictive performance of LODDS, LNR, and pN stage in OS and CSS was assessed by calculating C-index.

**Results:**

A total of 2,198 patients were included in this study. LODDS, LNR, and pN stage were associated with the OS and CSS of colorectal SRCC patients (all *P* < 0.05). LODDS showed a good performance in the OS (C-index: 0.704, 95% CI: 0.690–0.718), which was superior to LNR (C-index: 0.657, 95% CI: 0.643–0.671) and pN stage (C-index: 0.643, 95% CI: 0.629–0.657). The C-index of LODDS, LNR, and pN stage for CSS was 0.733 (95% CI: 0.719–0.747), 0.713 (95% CI: 0.697–0.729), and 0.667 (95% CI: 0.651–0.683), respectively.

**Conclusions:**

LODDS displayed a better predictive capacity in the OS and CSS than LNR and pN stage, indicating that LODDS may be effective to predict the prognosis of colorectal SRCC in the clinic.

## Introduction

Signet ring cell carcinoma (SRCC) is a rare and special subtype of colorectal cancer (CRC), accounting for approximately 1% of all CRC cases (1). SRCC, originating from undifferentiated stem cells of colorectal mucosa, is characterized by rapid development, poor differentiation, diffuse infiltration, and high metastatic rate (2, 3). Evidence has indicated that SRCC is an independent risk factor for the poor prognosis of CRC patients (4). Compared to patients with colorectal nonvariant adenocarcinoma, SRCC patients have a lower 5-year overall survival (OS) and cancer-specific survival (CSS) (1). Although there are many clinical studies on the prognosis of CRC, little attention has been paid to that of SRCC. Accurately estimating the prognosis of SRCC cases may help implement individualized treatment and select the optimal treatment strategy to increase the survival rate.

Lymph node metastasis (LNM) is an important risk factor for the poor prognosis and strongly affects therapeutic decisions in CRC (5). The 5-year OS ranges from 70% to 90% in CRC patients with negative lymph nodes (NLNs), while it ranges from 30% to 60% in patients with positive lymph nodes (PLNs) (6). The American Joint Committee on Cancer (AJCC) pathological nodal classification (pN) stratifies nodal involvement according to the number of involved lymph nodes and is regarded as the most important prognostic factor for CRC patients (6). The accuracy of pN stage is influenced by the number of examined lymph nodes (must be ≥12), and 48%–63% of cases have inadequate lymph node examination (7). This may lead to underestimated stages and improper treatment. To address the limitations of the pN stage system, lymph node ratio (LNR) is proposed, which is defined as the ratio of the number of PLNs to the total number of retrieved lymph nodes (TLNs) (8). LNR is less influenced by TLNs and has been reported as a prognostic factor in CRC (8, 9). However, for patients without LNM (pN0 patients), LNR cannot predict the prognosis better than pN stage system. Also, the number of NLNs significantly affects prognosis (10, 11). Both LNR and pN stage are not to consider the effect of NLNs.

Log odds of PLNs (LODDS), defined as the log of the ratio of the number of PLNs to the number of NLNs, has been introduced as a prognostic marker in rectal cancer (9). Xu et al. has reported that LODDS showed a good predictive capacity in the OS and CSS of gastric SRCC patients (12). In the study by Scarinci et al., LODDS was confirmed to be superior to LNR and pN stage in predicting the short-term OS of CRC (13). Also, Scarinci et al. suggested cohort studies with larger sample size to further explore whether it could be superior to LNR and pN stage in patients with subtypes (13). SRCC is characterized by high malignancy and poor prognosis and is a rare type of CRC to which less attention has been paid (1); thus, we further explore the association between LODDS and prognosis of colorectal SRCC patients and compare the predictive capacity of LODDS with pN stage system and LNR.

## Methods

### Study design and data source

This was a retrospective cohort study, and data were extracted from the Surveillance, Epidemiology and End Results (SEER) database (2004–2015, November 2018 submission), which was an authoritative source for cancer statistics in the United States (https://seer.cancer.gov/). SEER collected cancer incidence and survival data from 18 states and municipal registries, covering approximately 34.6% of the US population. SEER program was supported by the National Cancer Institute (NCI) and is freely available to the public. Therefore, informed consent from patients and approval from the Institutional Review Board of the Jiangyin Hospital of Traditional Chinese Medicine was not required for this study. All procedures involving human participants in this study were performed in accordance with the Declaration of Helsinki-2013 revision.

### Participants

Participants who met all the following criteria were included: (1) SRCC diagnosed in line with the International Classification of Disease for Oncology—third version (ICD-O-3; coded as 8490/3); (2) diagnosed as primary colorectal SRCC patients; (3) age at diagnosis ≥18 years; (4) with complete pathological information, operation, and complete survival data. Exclusion criteria were as follows: (1) missed data on lymph nodes; (2) with multiple primary tumors; (3) the reported diagnosis source from autopsy or death certificate or only clinically diagnosed.

### Data extraction

Data used in this study were extracted from the SEER 18 database (Nov 2018 Sub, 1975–2016 varying). The SEER*Stat 8.4.0 software was used to generate the case listing (https://seer.cancer.gov/data-software/documentation/seerstat/). We used data based on covariates (demographic characteristics, clinicopathological features, and treatment), prognostic variates (pN stage, LNR, and LODDS), and outcome variates (OS and CSS).

Demographic characteristics contained age (age at diagnosis), sex (male/female), race (white, black, other, unknown), marital status (single, married, unknown), and year of diagnosis (2004–2007, 2008–2011, 2012–2015). Clinicopathological characteristics contained tumor size (categorized by tertiles: <32, 32–64, >64 mm), primary site (cecum and appendix, colon, rectum), grade (I, well differentiated; II, moderately differentiated; III, poorly differentiated; IV, undifferentiated; and not stated), T stage (T1, T2, T3, and T4), and M stage (M0 and M1). Clinical characteristics contained the treatment (all assessed as categorical variables), chemotherapy (no/unknown and yes), radiation (no/unknown and yes), radiation with surgery (no/unknown, prior, and after/others), and surgery types (local/partial resection, total resection, and unspecific-underwent resection but no known the surgery site).

For comparative purposes, we classified lymph node status by pN stage, LNR, and LODDS. Based on AJCC eighth edition, pN stage was classified into N0 (no LNM), N1 (1–3 LNM), and N2 (4 and more LNM) (14). LNR was defined as the ratio between the number of PLNs and the number of TLNs, and divided into three groups: LNR 1 (≤0.32), LNR 2 (0.32–0.68), and LNR 3 (>0.68). LODDS value was calculated as follows: log_e_ (number of PLNs + 0.5)/(number of NLNs + 0.5), where 0.5 was added to avoid an infinite number (12). Patients were divided into 3 categories: LODDS 1 (≤−3.16), LODDS 2 (−3.16–0.60) and LODDS 3 (>0.60). The cut-off values of LNR and LODDS were evaluated by x-tile software (version 3.6.1, Yale University) based on minimum *P* value method (15).

Outcomes were OS and CSS. OS was defined as the period from diagnosis to death from any causes. CSS was defined as the duration from diagnosis to death attributed to SRCC. The assessment of OS and CSS was according to the records in the SEER database (alive, cancer-specific death, and other death). The follow-up was ended if patients died during the follow-up period.

### Statistical analysis

Categorical data were reported as number (*n*) and proportion (%), and compared using the Chi-square or nonparametric test. Continuous data in normal distribution were reported as mean ± standard deviation (mean ± SD) and compared using the independent-sample t test. Continuous data in skew distribution were expressed as median and quartile [M (Q1, Q3)] and compared using the independent-sample Wilcoxon rank sum test. Univariate Cox proportional hazard model (for OS) and univariate Cox competing risk model (for CSS) were used to identify the significant factors influencing OS and CSS. Association between pN stage, LNR, and LODDS with OS and CSS was explored using univariate and multivariate Cox proportional hazard model and Cox competing risk model, respectively, and results were shown as hazard ratio (HR) with 95% confidence interval (95% CI). Prediction capacity of the pN stage system, LNR, and LODDS in OS and CSS was evaluated by calculating the C-index. The prognostic capacity of the pN stage system, LNR, and LODDS was also assessed according to the number of retrieved lymph nodes (TLNs < 12 or TLNs ≥ 12) and neoadjuvant radiotherapy (with or without). Statistical analyses were performed using SPSS 26.0 software for Mac (IBM, Armonk, NY, United States). *P* <0.05 was considered statistically significant.

## Results

### Patients’ selection and characteristics

**Figure 1:**
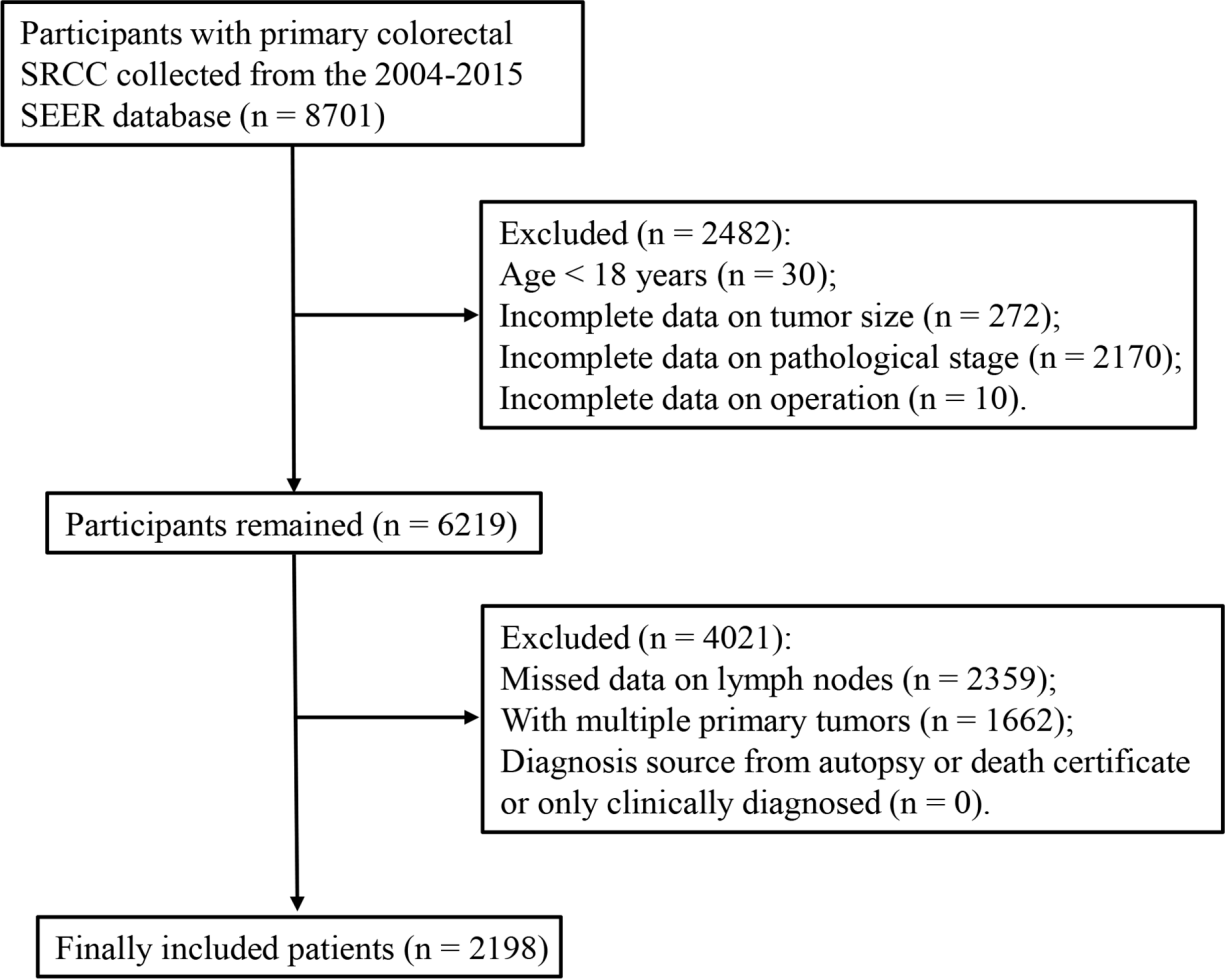
The flowchart of selection of patients.

We extracted 8,701 patients with primary colorectal SRCC from the SEER database. Of these, 2,482 patients were excluded because they were younger than 18 years (*n* = 30) and had incomplete data on tumor size (*n* = 272), pathological stage (*n* = 2170), and the operation (*n* = 10). Of the remaining 6,219 patients, we further excluded 4,021 patients due to missing data on lymph nodes (*n* = 2,359), with multiple primary tumors (*n* = 1,662), and with diagnosis source from autopsy or death certificate or only clinically diagnosed (*n* = 0). Finally, a total of 2198 colorectal SRCC patients were included in our study (Figure 1). These patients were composed of 1,101 men (50.09%) and 1,097 women (49.91%), and the mean age was 63.47 ± 16.29 years. The median survival time of alive patients, patients with SRCC-specific death, and patient died from other causes was 56 months (range 27–96), 13 months (range 6–24), and 20 months (range 5.00–58.00), respectively. Age, year of diagnosis, tumor size categories, primary site, AJCC T stage, AJCC N stage, AJCC M stage, LNR categories, LODDS categories, chemotherapy, surgery types, and survival months were significantly different among the groups. Characteristics of the included patients are summarized in Table 1.

**Table 1:** Characteristics of the included patients.

Variables	Total	Alive (*n* = 703)	SRCC-specific deaths (*n* = 1238)	Other deaths (*n* = 257)	Statistics	*P*
Age (years), Mean ± SD	63.47 ± 16.29	60.96 ± 15.58	62.46 ± 16.36	75.19 ± 12.59	*F* = 83.025	<0.001
Sex, *n* (%)					*χ*^2^ = 3.796	0.150
Male	1,101 (50.09)	344 (48.93)	640 (51.70)	117 (45.53)		
Female	1,097 (49.91)	359 (51.07)	598 (48.30)	140 (54.47)		
Race, *n* (%)					χ^2^ = 4.556	0.602
White	1,824 (82.98)	587 (83.50)	1,016 (82.07)	221 (85.99)		
Black	185 (8.42)	58 (8.25)	108 (8.72)	19 (7.39)		
Other	183 (8.33)	55 (7.82)	112 (9.05)	16 (6.23)		
Unknown	6 (0.27)	3 (0.43)	2 (0.16)	1 (0.39)		
Marital status, *n* (%)					χ^2^ = 4.276	0.370
Single	372 (16.92)	111 (15.79)	226 (18.26)	35 (13.62)		
Married	1,742 (79.25)	565 (80.37)	966 (78.03)	211 (82.10)		
Unknown	84 (3.82)	27 (3.84)	46 (3.72)	11 (4.28)		
Year of diagnosis, *n* (%)					χ^2^ = 125.878	<0.001
2004–2007	744 (33.85)	159 (22.62)	463 (37.40)	122 (47.47)		
2008–2011	711 (32.35)	199 (28.31)	428 (34.57)	84 (32.68)		
2012–2015	743 (33.80)	345 (49.08)	347 (28.03)	51 (19.84)		
Tumor size categories (mm), *n* (%)					χ^2^ = 24.848	<0.001
<32	735 (33.44)	284 (40.40)	363 (29.32)	88 (34.24)		
32–64	759 (34.53)	217 (30.87)	453 (36.59)	89 (34.63)		
>64	704 (32.03)	202 (28.73)	422 (34.09)	80 (31.13)		
Primary site, *n* (%)					χ^2^ = 22.505	<0.001
Cecum and appendix	809 (36.81)	229 (32.57)	501 (40.47)	79 (30.74)		
Colon	1,090 (49.59)	381 (54.20)	561 (45.32)	148 (57.59)		
Rectum	299 (13.60)	93 (13.23)	176 (14.22)	30 (11.67)		
Grade, *n* (%)					χ^2^ = 11.708	0.165
I	19 (0.86)	9 (1.28)	7 (0.57)	3 (1.17)		
II	123 (5.60)	52 (7.40)	56 (4.52)	15 (5.84)		
III	1,578 (71.79)	488 (69.42)	902 (72.86)	188 (73.15)		
IV	309 (14.06)	100 (14.22)	173 (13.97)	36 (14.01)		
Not stated	169 (7.69)	54 (7.68)	100 (8.08)	15 (5.84)		
AJCC T stage, *n* (%)					χ^2^ = 234.881	<0.001
T1	52 (2.37)	38 (5.41)	7 (0.57)	7 (2.72)		
T2	81 (3.69)	52 (7.40)	15 (1.21)	14 (5.45)		
T3	1,071 (48.73)	424 (60.31)	501 (40.47)	146 (56.81)		
T4	994 (45.22)	189 (26.88)	715 (57.75)	90 (35.02)		
AJCC N stage, *n* (%)					χ^2^ = 359.574	< 0.001
N0	472 (21.47)	280 (39.83)	102 (8.24)	90 (35.02)		
N1	500 (22.75)	185 (26.32)	257 (20.76)	58 (22.57)		
N2	1,226 (55.78)	238 (33.85)	879 (71.00)	109 (42.41)		
AJCC M stage, *n* (%)					χ^2^ = 294.888	<0.001
M0	1,517 (69.02)	632 (89.90)	671 (54.20)	214 (83.27)		
M1	681 (30.98)	71 (10.10)	567 (45.80)	43 (16.73)		
LNR categories, *n* (%)					χ^2^ = 363.152	<0.001
LNR 1	1,098 (49.95)	527 (74.96)	409 (33.04)	162 (63.04)		
LNR 2	489 (22.25)	118 (16.79)	330 (26.66)	41 (15.95)		
LNR 3	611 (27.80)	58 (8.25)	499 (40.31)	54 (21.01)		
LODDS categories, *n* (%)					χ^2^ = 416.360	<0.001
LODDS 1	376 (17.11)	248 (35.28)	66 (5.33)	62 (24.12)		
LODDS 2	1,176 (53.50)	390 (55.48)	648 (52.34)	138 (53.70)		
LODDS 3	646 (29.39)	65 (9.25)	524 (42.33)	57 (22.18)		
Chemotherapy, *n* (%)					χ^2^ = 102.472	<0.001
No/unknown	951 (43.27)	292 (41.54)	473 (38.21)	186 (72.37)		
Yes	1,247 (56.73)	411 (58.46)	765 (61.79)	71 (27.63)		
Radiation, *n* (%)					χ^2 ^= 5.014	0.082
No/unknown	1,967 (89.49)	633 (90.04)	1,095 (88.45)	239 (93.00)		
Yes	231 (10.51)	70 (9.96)	143 (11.55)	18 (7.00)		
Radiation sequence with surgery, *n* (%)					χ^2^ = 6.359	0.174
No/unknown	1,967 (89.49)	633 (90.04)	1,095 (88.45)	239 (93.00)		
Prior	120 (5.46)	40 (5.69)	70 (5.65)	10 (3.89)		
After/others	111 (5.05)	30 (4.27)	73 (5.90)	8 (3.11)		
Surgery types, *n* (%)					χ^2^ = 10.892	0.028
Local/partial	2,089 (95.04)	672 (95.59)	1,168 (94.35)	249 (96.89)		
Total	98 (4.46)	25 (3.56)	67 (5.41)	6 (2.33)		
Unspecific	11 (0.50)	6 (0.85)	3 (0.24)	2 (0.78)		
Survival months, M (Q_1_, Q_3_)	20.00 (9.00, 47.00)	56.00 (27.00, 96.00)	13.00 (6.00, 24.00)	20.00 (5.00, 58.00)	χ^2^ = 652.945	<0.001

SRCC, signet ring cell carcinoma; AJCC, American Joint Committee on Cancer; LNR, lymph node ratio; LODDS, log odds of positive lymph nodes.

### The association between pN stage, LNR, and LODDS with the OS and CSS of colorectal SRCC patients

Supplementary Table S1 shows that age, married status, tumor size, AJCC T stage, AJCC M stage, chemotherapy, and primary site were significant factors affecting the OS of colorectal SRCC patients. Marriage status, tumor size, AJCC T stage, AJCC M stage, chemotherapy, surgery types, and primary site were significant factors affecting the CSS of colorectal SRCC patients. Considering many studies have reported radiotherapy as an important influencing factor of the prognosis in colorectal SRCC (16, 17), radiation sequence with surgery was also considered as a covariate in this study.

In the univariate analysis, higher pN stage, LNR, and LODDS were associated with the worse OS and CSS (all *P* < 0.001). After adjusting for age, married status, tumor size, AJCC T stage, AJCC M stage, chemotherapy, primary site, and radiation sequence with surgery, pN stage (N1: HR = 2.04, 95% CI: 1.69–2.46; N2: HR = 3.40, 95% CI: 2.86–4.03), LNR (LNR 2: HR = 1.96, 95% CI: 1.72–2.26; LNR 3: HR = 2.97, 95% CI: 2.60–3.39), and LODDS (LODDS 2: HR = 2.51, 95% CI: 2.06–3.04; LODDS 3: HR = 5.08, 95% CI: 4.13–6.26) showed similar results in the OS. Also, N1, N2, LNR 2, LNR 3, LODDS 2, and LODDS 3 were associated with higher risk of worse CSS after adjusting for married status, tumor size, AJCC T stage, AJCC M stage, chemotherapy, surgery types, primary site, and radiation sequence with surgery, with HR of 2.46 (95% CI: 1.94–3.10), 3.90 (95% CI: 3.11–4.87), 2.02 (95% CI: 1.74–2.34), 2.70 (95% CI: 2.33–3.13), 3.21 (95% CI: 2.48–4.15), and 5.64 (95% CI: 4.29–7.43), respectively (Table 2).

**Table 2:** Association of pN stage, LNR, and LODDS with OS and CSS in colorectal SRCC patients.

Variables	OS				CSS			
	Univariate		Multivariate^a^		Univariate		Multivariate^b^	
	HR (95% CI)	*P*	HR (95% CI)	*P*	HR (95% CI)	*P*	HR (95% CI)	*P*
AJCC N stage								
N0	Ref		Ref		Ref		Ref	
N1	1.96 (1.63–2.34)	<0.001	2.04 (1.69–2.46)	<0.001	2.93 (2.34–3.68)	<0.001	2.46 (1.94–3.10)	<0.001
N2	3.57 (3.05–4.18)	<0.001	3.40 (2.86–4.03)	<0.001	5.45 (4.45–6.68)	<0.001	3.90 (3.11–4.87)	<0.001
LNR categories								
LNR 1	Ref		Ref		Ref		Ref	
LNR 2	2.16 (1.89–2.46)	<0.001	1.96 (1.72–2.26)	<0.001	2.52 (2.19–2.91)	<0.001	2.02 (1.74–2.34)	<0.001
LNR 3	3.57 (3.16–4.02)	<0.001	2.97 (2.60–3.39)	<0.001	3.92 (3.44–4.48)	<0.001	2.70 (2.33–3.13)	<0.001
LODDS categories								
LODDS 1	Ref		Ref		Ref		Ref	
LODDS 2	2.61 (2.17–3.15)	<0.001	2.51 (2.06–3.04)	<0.001	3.99 (3.11–5.13)	<0.001	3.21 (2.48–4.15)	<0.001
LODDS 3	5.94 (4.90–7.21)	<0.001	5.08 (4.13–6.26)	<0.001	8.95 (6.93–11.57)	<0.001	5.64 (4.29–7.43)	<0.001

OS, overall survival; CSS, cancer-specific survival; HR, hazard ratio; CI, confidence interval; AJCC, American Joint Committee on Cancer; LNR, lymph node ratio; LODDS, log odds of positive lymph nodes.

^a^ Multivariate: Analysis adjusted age, married status, tumor size, AJCC T stage, AJCC M stage, chemotherapy, primary site, and radiation sequence with surgery.

^b^ Multivariate: Analysis adjusted married status, tumor size, AJCC T stage, AJCC M stage, chemotherapy, surgery types, primary site, and radiation sequence with surgery.

### Predictive capacity of pN stage, LNR, and LODDS in the OS and CSS of colorectal SRCC patients

Table 3 shows the predictive performance of pN stage, LNR, and LODDS in the OS and CSS. LODDS (C-index: 0.704, 95% CI: 0.690–0.718) had a better performance in predicting the OS than LNR (C-index: 0.657, 95% CI: 0.643–0.671) and pN stage (C-index: 0.643, 95% CI: 0.629–0.657). We also found the better performance of LODDS in the CSS, with C-index of 733 (95%CI: 0.719–0.747), which was higher than 0.713 (95% CI: 0.697–0.729) of LNR and 0.667 (95% CI: 0.651–0.683) of pN stage.

**Table 3:** Predictive capacity of pN stage, LNR, and LODDS in the OS and CSS of colorectal SRCC patients.

Variables	OS	CSS
	C-index (95% CI)	C-index (95% CI)
LODDS	0.704 (0.690–0.718)	0.733 (0.719–0.747)
LNR	0.657 (0.643–0.671)	0.713 (0.697–0.729)
pN	0.643 (0.629–0.657)	0.667 (0.651–0.683)

OS, overall survival; CSS, cancer-specific survival; CI, confidence interval; LNR, lymph node ratio; LODDS, log odds of positive lymph nodes.

Table 4 displays the predictive capacity of pN stage, LNR, and LODDS in the OS and CSS based on retrieved lymph nodes. In patients with number of retrieved lymph nodes <12, LODDS had a better predictive capacity in the OS and CSS, with C-index of 0.663 (95% CI: 0.641–0.691) and 0.711 (95% CI: 0.681–0.741), respectively. In patients with number of retrieved lymph nodes ≥12, the results remained similar, with C-index of 0.713 (95% CI: 0.701–0.731) for OS and 0.739 (95% CI: 0.721–0.751) for CSS.

**Table 4:** Prognostic efficacy of pN stage, LNR, and LODDS in the OS and CSS of colorectal SRCC patients based on retrieved lymph nodes.

Variables	TLNs < 12		TLNs ≥ 12	
	OS	CSS	OS	CSS
	C-index (95% CI)	C-index (95% CI)	C-index (95% CI)	C-index (95% CI)
LODDS	0.663 (0.641–0.691)	0.711 (0.681–0.741)	0.713 (0.701–0.731)	0.739 (0.721–0.751)
LNR	0.621 (0.591–0.651)	0.676 (0.651–0.711)	0.666 (0.651–0.681)	0.725 (0.711–0.741)
pN	0.623 (0.591–0.651)	0.656 (0.631–0.691)	0.660 (0.641–0.681)	0.684 (0.671–0.701)

OS, overall survival; CSS, cancer-specific survival; CI, confidence interval; LNR, lymph node ratio; LODDS, log odds of positive lymph nodes; TLNs, total number of retrieved lymph nodes.

## Discussion

The prognosis of CRC has gained much attention (18, 19), but few studies explored the prognosis of colorectal SRCC, which is a rare and special subtype of CRC and characterized by a poorer prognosis (1). In this study, we found that LODDS, LNR, and pN stage were associated with the OS and CSS of colorectal SRCC patients. LODDS had a better performance to predict the OS and CSS than LNR and pN stage in colorectal SRCC patients. Similar results were found in patients with TLNs either <12 or ≥12.

LNM is an important factor affecting the prognosis of CRC and affects therapeutic decisions (5). AJCC pN stage and LNR are both reported as prognostic factors in CRC (6, 8), but AJCC pN stage is limited by TLNs and LNR did not consider the effect of NLNs (7, 11). LODDS has been reported to be less influenced by TLNs and takes into account the number of NLNs (9). Scarinci et al. have reported the association of LODDS, LNR, and pN stage with the OS in CRC (13). Similarly, our study has found that LODDS, LNR, and pN stage were associated with OS and CSS of colorectal SRCC patients.

Several studies have shown the strong prognostic ability of LODDS in SRCC (12, 20). In a cohort study, Wang et al. found that, compared with the pN stage, LODDS was a highly reliable index with a good predictive performance for the OS and CSS of patients with esophageal SRCC (20). Xu et al. enrolled 1,365 patients with gastric SRCC and developed a prognostic nomogram based on LODDS, which provided a more satisfying predictive capacity both in the OS and CSS than AJCC TNM stage alone (12). Scarinci et al. has reported that the predictive performance of LODDS was superior to LNR and pN stage in the OS of CRC patients and suggested a future study to verify their findings in the subtype of CRC (13). In this study, we found that LODDS outmatched the pN stage and LNR for the prediction of OS and CSS in colorectal SRCC, which made up the gap of the study reported by Scarinci et al. These findings clearly showed that LODDS could accurately predict the survival of colorectal SRCC patients. Therefore, LODDS may be a useful and scientific tool to evaluate lymph node dissection and to consider ratio-based index into the prognosis of colorectal SRCC patients and was recommended to examine LNM in practical use.

Some observational studies have found that adequate evaluation of TLNs (≥12) is associated with the increased survival (21–23). However, half of the CRC patients receive inadequate lymph node assessment (7). Herein, we performed subgroup analysis to explore the prediction capacity of LODDS, LNR, and pN based on the TLNs. In colorectal SRCC patients with TLNs < 12, the predictive performance of LODDS was superior to LNR and pN stage in the OS and CSS. We found similar results in the patients with TLNs ≥12. Our findings further confirmed the accuracy of LODDS in the prediction of OS and CSS.

There are some strengths in our study. First, our data are extracted from the SEER database, which contains a large, validated, representative sample of the US population. Second, our study makes up for the gap in the prediction capacity of LODDS in colorectal SRCC, which indicates that LODDS may be a useful and scientific tool to evaluate lymph node dissection and predict the prognosis of colorectal SRCC patients. In addition, our study has some limitations. First, this is a retrospective study, which may cause selection bias and information bias. Second, important factors associated with the prognosis of colorectal SRCC, such as pathological data, nutritional status, and lifestyle, were not recorded in the SEER database. Third, neoadjuvant therapy may affect postoperative LNMs, but chemotherapy time is not recorded and radiotherapy sequence of most patients (89.49%) was recorded as no/unknown in the database. Therefore, patients undergoing neoadjuvant therapy cannot be accurately distinguished, which limits us to explore the effect of neoadjuvant therapy on the results. However, we adjust the relevant information on chemotherapy and radiotherapy to minimize its impact on the results. Fourth, external validation is not performed due to the lack of a sufficient sample size; further studies in the actual clinical samples are needed to validate our findings.

## Conclusion

Our study found the better predictive capacity of LODDS than LNR and pN stage in the OS and CSS in colorectal SRCC, indicating that LODDS may be a useful and scientific tool to assess the prognosis of colorectal SRCC patients in the clinic.

## Data Availability

Publicly available datasets were analyzed in this study. This data can be found here: SEER database, https://seer.cancer.gov/.
