# Supplementary material for: Prognostic value of log odds of positive lymph nodes, lymph node ratio, and N stage in patients with colorectal signet ring cell carcinoma: A retrospective cohort study
Source: Front Surg. 2023 Jan 5;9:1019454. doi: 10.3389/fsurg.2022.1019454 (PMC9849566; doi:10.3389/fsurg.2022.1019454)
Supplement: Supplementary file 1 [file Table1.docx]

**Supplementary Table S1 Univariable analysis for screening survival-related factors**

| Variables | OS | | CSS | |
| --- | --- | --- | --- | --- |
|  | HR (95% CI) | *P* | HR (95% CI) | *P* |
| Age | 1.01 (1.01-1.01) | < 0.001 | 0.99 (0.99-1.01) | 0.058 |
| Sex |  |  |  |  |
| Male | Ref |  | Ref |  |
| Female | 0.98 (0.89-1.09) | 0.706 | 0.94 (0.84-1.05) | 0.271 |
| Race |  |  |  |  |
| White | Ref |  | Ref |  |
| Black | 1.17 (0.97-1.40) | 0.098 | 1.11 (0.92-1.35) | 0.262 |
| Other | 1.06 (0.88-1.27) | 0.546 | 1.10 (0.92-1.32) | 0.314 |
| Unknown | 0.78 (0.25-2.44) | 0.671 | 0.70 (0.18-2.73) | 0.606 |
| Marital status |  |  |  |  |
| Single | Ref |  | Ref |  |
| Married | 0.85 (0.75-0.98) | 0.019 | 0.83 (0.72-0.96) | 0.012 |
| Unknown | 0.92 (0.69-1.23) | 0.577 | 0.85 (0.62-1.15) | 0.291 |
| Tumor size |  |  |  |  |
| < 32 mm | Ref |  | Ref |  |
| 32-64 mm | 1.38 (1.21-1.56) | < 0.001 | 1.39 (1.22-1.59) | < 0.001 |
| > 64 mm | 1.46 (1.28-1.65) | < 0.001 | 1.48 (1.29-1.70) | < 0.001 |
| Grade |  |  |  |  |
| I | Ref |  | Ref |  |
| II | 1.13 (0.58-2.19) | 0.718 | 1.30 (0.61-2.74) | 0.494 |
| III | 1.67 (0.89-3.10) | 0.109 | 1.93 (0.95-3.90) | 0.068 |
| IV | 1.81 (0.96-3.42) | 0.066 | 2.02 (0.99-4.15) | 0.054 |
| Not stated | 1.58 (0.83-3.01) | 0.166 | 1.95 (0.94-4.02) | 0.073 |
| AJCC T stage |  |  |  |  |
| T1 | Ref |  | Ref |  |
| T2 | 1.41 (0.74-2.67) | 0.292 | 1.40 (0.58-3.37) | 0.458 |
| T3 | 3.19 (1.88-5.41) | < 0.001 | 4.54 (2.18-9.45) | < 0.001 |
| T4 | 6.25 (3.68-10.62) | < 0.001 | 9.35 (4.50-19.41) | < 0.001 |
| AJCC M stage |  |  |  |  |
| M0 | Ref |  | Ref |  |
| M1 | 3.00 (2.69-3.34) | < 0.001 | 3.30 (2.95-3.70) | < 0.001 |
| Chemotherapy |  |  |  |  |
| No/unknown | Ref |  | Ref |  |
| Yes | 0.82 (0.74-0.91) | < 0.001 | 1.16 (1.03-1.31) | 0.014 |
| Radiation |  |  |  |  |
| No/unknown | Ref |  | Ref |  |
| Yes | 0.86 (0.73-1.01) | 0.073 | 0.99 (0.85-1.15) | 0.892 |
| Radiation sequence with surgery |  |  |  |  |
| No/unknown | Ref |  | Ref |  |
| Prior | 0.80 (0.64-1.00) | 0.053 | 1.02 (0.75-1.40) | 0.890 |
| After/others | 0.93 (0.74-1.16) | 0.503 | 0.79 (0.55-1.12) | 0.190 |
| Surgery types |  |  |  |  |
| Local/partial | Ref |  | Ref |  |
| Total | 1.18 (0.93-1.49) | 0.177 | 1.32 (1.06-1.66) | 0.015 |
| Unspecific | 0.57 (0.24-1.37) | 0.209 | 0.39 (0.13-1.18) | 0.097 |
| Primary site |  |  |  |  |
| Cecum & appendix | Ref |  | Ref |  |
| Colon | 0.84 (0.76-0.94) | 0.002 | 0.77 (0.68-0.87) | < 0.001 |
| Rectum | 0.81 (0.69-0.95) | 0.008 | 0.81 (0.69-0.95) | 0.010 |

OS, overall survival; CSS, cancer-specific survival; HR, hazard ratio; CI, confidence interval; AJCC, American Joint Committee on Cancer.
